# Therapeutic endobronchial resection of a benign tumor in a patient with cystic fibrosis

**DOI:** 10.1002/ccr3.2325

**Published:** 2019-08-22

**Authors:** Sophie Gohy, Delphine Hoton, Antoine Froidure

**Affiliations:** ^1^ Department of Pulmonology Cliniques Universitaires Saint‐Luc, UCL Brussels Belgium; ^2^ Cystic Fibrosis Reference Center Cliniques Universitaires Saint‐Luc, UCL Brussels Belgium; ^3^ Department of Pathology Cliniques Universitaires Saint‐Luc, UCL Brussels Belgium

**Keywords:** cystic fibrosis, endobronchial polyp, endobronchial resection, rigid bronchoscopy

## Abstract

This report highlights the usefulness of bronchoscopy in case of recurrent pneumonia with the same localization even in CF patients where the presence of bronchiectasis as promoting factor of infections could delay the diagnosis.

## 1 INTRODUCTION

A patient with cystic fibrosis presented recurrent pneumonia in the upper right lobe. A polypoid lesion was found during bronchoscopy. We report the first case of a successful endoscopic resection of an inflammatory polyp without need for open surgery and without recurrence of the tumor nor lung infection.

## 2 CASE REPORT

A 41‐year‐old female with cystic fibrosis (CF) (F508del/3272‐26A → G), pancreatic sufficient with normal body mass index (22.5 kg/m^2^) and severe lung disease (forced expiratory volume in one second [FEV1], 34% of predicted values), was admitted for sudden onset of high‐grade fever, respiratory‐dependent anterior chest pain and cough worsening. Anterior right crackles were present at auscultation. She had a past medical history of three pneumonia in the upper right lobe within the last 18 months (no other significant past medical history excluding CF). Her treatment comprised daily respiratory physiotherapy, inhaled steroids, long‐acting beta‐agonist, dornase alpha, inhaled colomycin and vitamin D. Regular courses of ciprofloxacin, minocycline, or trimethoprim/sulfamethoxazole were prescribed for the presence of *Pseudomonas aeruginosa*, *Stenotrophomonas maltophilia* and *Staphylococcus aureus* in the expectorations.

**Figure 1:**
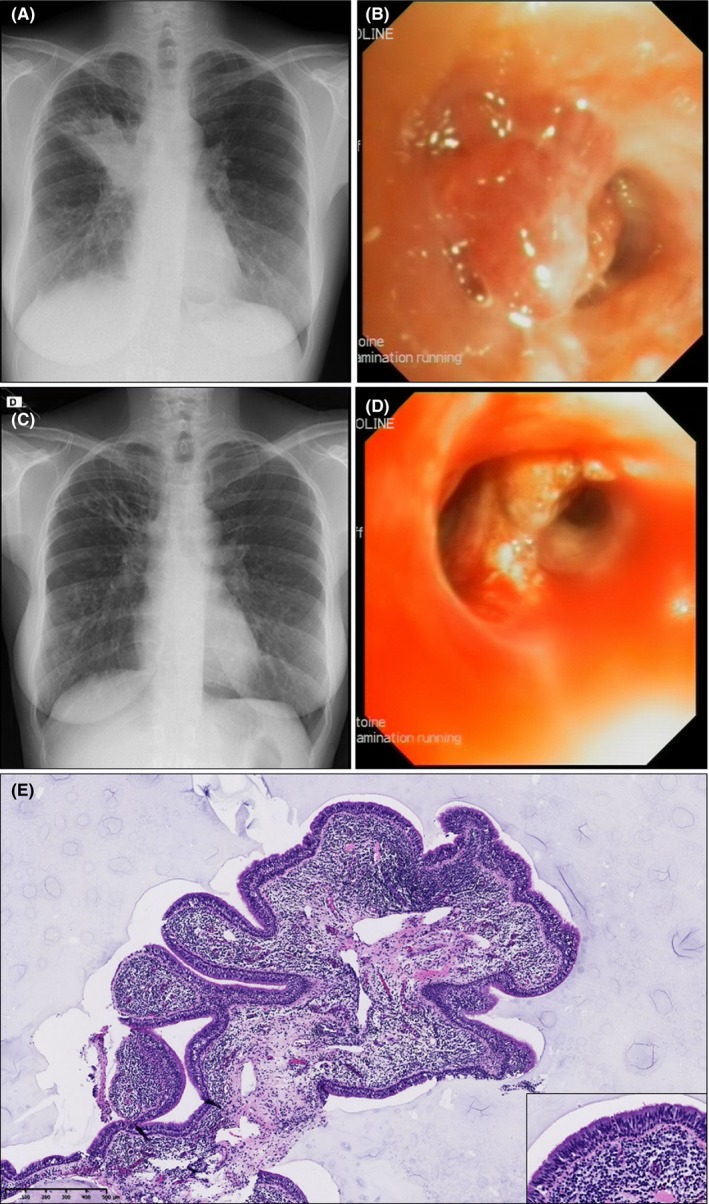
Radiological, endoscopic, and histological diagnosis of a right anterior segment obstruction (B3) due to endobronchial inflammatory polyps. A, Chest X‐Ray showing an anterior segment (B3) consolidation of the right upper lobe. B, Endobronchial polypoid lesion obstructing right anterior bronchus of the upper lobe (RB3). C, Chest X‐Ray showing the regression of the right anterior segment (B3) consolidation of the upper lobe after therapeutic bronchoscopy. D, Endobronchial polypoid lesion removed during rigid bronchoscopy by cryotherapy and argon plasma coagulation. E, Photomicrograph of polyps showing an exophitic lesion with a normal pseudostratified respiratory epithelium and a fibrovascular stroma containing inflammatory cells. Scale bar, 500 µm

Initial work‐up showed a right anterior segment (B3) consolidation on the chest X‐Ray (Figure 1A) along with a marked systemic inflammation (white blood cells: 21 230/µL, neutrophils: 17 230/µL, C‐reactive protein: 81 mg/L). The patient was admitted for intravenous antibiotics (tobramycin and meropenem). Due to the recurrence of pneumonia in the same lobe, a flexible bronchoscopy was performed during the hospitalization and revealed an obstruction of the right antero‐superior bronchi (RB3) by an endobronchial polypoid lesion (Figure 1B). Pathological examination of the lesion biopsies demonstrated a fibrovascular and inflammatory (mainly mononuclear cells and some neutrophils) stroma covered by a pseudostratified respiratory epithelium, altogether consistent with benign polyp. After 14 days of intravenous antibiotics, the patient improved clinically, biologically, and functionally with FEV1 at 42% of predicted values. She was discharged on her previous treatment. Given the previous three pneumonia in the same segment and in order to prevent further recurrence, it was decided to remove the tumor endoscopically one‐month later (in stable conditions). The procedure was performed under general anesthesia and with jet‐ventilation (high frequency, low pressure) to prevent barotrauma. Through a rigid bronchoscope, we used cryotherapy and argon plasma coagulation to remove the tumor. After the procedure, the right antero‐superior bronchi (RB3) was freed and the chest X‐Ray clearly improved (Figure 1C,D). Pathological analyses confirmed the diagnosis of inflammatory polyps containing mainly mononuclear inflammatory cells and neutrophils (Figure 1E). The lung function remained stable and neither recurrence of the tumor nor lung infection was observed during a 10‐month follow‐up.

## 3 DISCUSSION

In this case, we report successful removal of an endoluminal polyp, responsible for multiple recurrences of lower respiratory tract infections in a CF patient. The presence of the polyp was demonstrated through a flexible bronchoscopy, as CT‐scan failed to clearly demonstrate airway obstruction. Afterward, elective endoscopic removal of the lesion, under general anesthesia and through a rigid bronchoscope, prevented recurrence of pneumonia, without any consequence on pulmonary lung function tests.

Inflammatory endobronchial polyps account for 4%‐19% of benign trachea‐bronchial tumors.^1^, ^2^ In cystic fibrosis, polyps have so far only been described after lung transplant or following bilobectomy for recurrent chest infections (multiple small lesions that were not identified on preoperative chest CT).^3^, ^4^ The diagnosis is often challenging.^5^ Inflammatory polyps may regress spontaneously but are classically resected to confirm the benign condition and/or to treat symptomatic patients that experience relapsing respiratory infections, haemoptysis, and atelectasis. Endoscopic resection is the treatment of choice for symptomatic lesion to avoid open surgery.^6^ Bronchial polyposis is supposedly related to chronic infection and inflammation like in CF or tuberculosis, nontuberculosis pulmonary infection, and cytomegalovirus infection. Occurrence of polyps has been described in asthma, following exposure to chronic smoke inhalation, after mechanical ventilation in children. A polyp may also appear following transbronchial needle aspiration or as a reaction toward a foreign body. Treating the underlying cause may lead to the regression of the lesion, while evidence regarding corticosteroid therapy is scarce. Similarly to what has been described in the nasal polyps in CF, respiratory epithelium is surrounded by a fibrovascular stroma containing inflammatory cells that are mainly neutrophils.

In conclusion, this case report illustrates that endoluminal polyps are a rare form of benign lesion potentially affecting CF patients. Flexible bronchoscopy should be performed in CF patients suffering from recurrent infections with the same localization. Finally, endoscopic removal of benign tumor is possible, effective and safe, even with a severe CF‐lung disease and without subsequent worsening of the lung function worsening.

## CONFLICT OF INTEREST

None declared.

## AUTHOR CONTRIBUTION

SG and AF wrote the paper, DH reviewed the paper.
